# Significant increase of firework induced eye injuries in Germany and The Netherlands- are we doing enough to protect minors and bystanders?

**DOI:** 10.1007/s00417-024-06677-6

**Published:** 2024-12-04

**Authors:** Ameli Gabel-Pfisterer, Stefan Johann Lang, Daniel Boehringer, Hansjürgen Agostini, Lotte C. de Geus, Jan Tjeerd de Faber, the German firework study group, M. Fuest, P. Walter, I. Winkelmann, K. Hartmann, C. Kojetinsky, A. Mueller, C. Dempe, N. Al-Ashi, H. Breuß, I. Seibel, M. Gutmann, T. Bonaventura, O. Zeitz, B. Müller, A. Joussen, P. Schwarz, Ch Wirbelauer, H. Hofmayer, J. Wachtlin, N. I. Steinhorst, J. F. Meyer, B. Acar, S. Aisenbrey, K. Goebel, P. Rieck, M. Shalabi, M. Tohami, E. C. Thieme, A. Walch, J. Verbeck, M. Alnawaiseh, T. Schultz, N. Tsiampalis, J. Rehmann, U. Sliwowska, M. Schojai, K. Schulze, N. Kamguia, C. Wirtz, B. H. Dick, Y. Liermann, F. M. Schuetzeichel, D. Voelcker, M. Wintergerst, M. Pfau, C. Melzer, D. Hoegen, F. Bosch, J. C. Andresen, T. Krohne, F. Holz, A. Alahmad, M. Kathke, A. Sturm, E. Chankiewitz, O. Kemper, A. Heider, B. Erdogan-Uelker, N. Krainau, M. Thiele, S. Brandtner, J. Hecker, P. Strassburger, K. Engelmann, F. Lehmann, U. Brunner, H. Sachs, S. Koerner, L. Grajewski, L. Krause, W. Bayoudh, B. Yanar, D. Mpoutsis-Voutsis, J. Gerhardt, K. Rüdiger, T. Böker, T. Hejna, J. Hejna, K. Kiel, B. Breuer, E. Matthe, D. Sandner, L. Pillunat, J. Prueß-Hölscher, R. Kourukmas, M. Cieplucha, G. Geerling, R. A. Widder, G. Roessler, K. Bronikowska, M. Thomalla, F. Filev, B. von Jagow, J. Walther, C. Zollfrank, M. Blum, T. Theofilos, Y. M. Christian, E. K. Friedrich, A. Foerster, I. Diamantis, C. Braun, T. Kiefer, P. Rating, M. Fiorentzis, N. E. Bechrakis, A. Scheider, K. P. Kaiser, M. L. Biller, J. Bucur, T. Arad, M. Müller, T. Kohnen, T. J. Grabs, U. Vossmerbaeumer, C. Puk, Y. Laich, T. Reinhard, J. Quandt, A. Lieder, J. Seewald, C. Mais, R. E. Carlos, M. H. Graef, M. Rehak, J. Schrecker, C. Schinzel, S. T. Meyer, N. Feltgen, H. Hoerauf, T. Trzos, I. Prusiecki, M. C. Bründer, S. Paul, A. Stahl, R. Wienrich, A. Viestenz, A. Huth, A. Viestenz, A. Schulz, B. Fuisting, L. Mautone, A. Özen, N. Kaupke, L. Kröger, M. Alsarrani, I. Lau, J. Birtel, F. Hagenau, J. Wildner, N. Kounatidou, A. Hassenstein, C. Skevas, V. Knospe, S. Vardanyan, C. Grohmann, M. Spitzer, L. Fuhrmann, M. Schargus, M. T. Eddy, D. Rose, K. Reinkemeier, L. Armonies, B. Stemplewitz, U. Schaudig, B. Book, K. Hufendiek, E. Panidou-Marschelke, E. Sinicin, M. Lindziute, J. T. R. Rauscher, M. Hamann, C. Framme, A. Scheuerle, M. Auerbach, C. Beisse, K. Rohrschneider, R. Khoramnia, G. Auffahrt, N. Mala, L. Hesse, A. Sneyers, P. Kohlhas, E. Flockerzi, F. N. Fries, L. Daas, B. Seitz, R. Augsten, M. Aghi, M. Zankel, A. Ghaith, S. Weber, U. Voigt, D. Meller, J. O. Rudolph, M. Müller, F. Brede, M. Alia, F. Treumer, M. Saeger, B. Nölle, C. Ehlken, J. B. Roider, A. Hueber, C. Cursiefen, N. Schrage, P. Esser, M. Kroeger, N. Viehweg, M. Knorr, P. Meier, C. Girbardt, C. Bormann, N. Suckert, J. Letzel, F. Ziemssen, V. Pawlik, C. Schiemenz, M. Busch, P. Schubart, R. Piria, M. Stöcker, A. M. Mohi Sefat, F. Rommel, S. Grisanti, I. Bastron, M. Benthami, D.-N. Roman, S. Kaskel-Paul, C. Argyrios, A. Agharza, K. Strobel Bermond, L. O. Hattenbach, M. Erwemi, F. Schlichtenbrede, B. Stoffelns, A. Schuster, N. Pfeiffer, C. Paul, W. Sekundo, G. Renieri, H. Thieme, P. Foerster, S. Priglinger, E. Von Koskull, M. Maier, F. Alten, N. Eter, K. C. Brinkmann, F. Alshikh, V. Klishko, U. Holland, A. Medra, A. Weber, H. Höh, F. Luciani, J. Schmidbauer, A. Pielen, P. C. Horn, K. Hille, S. Grafmueller, G. Esper, T. Ahmels, L. Kolbeck, P. Kupper, A.-S. Schröder, F. Keller, S. Schrader, F. Höhn, M. G. Häringer, J. Schiemann, A. Liekfeld, M. Dütsch, V. Schnitzbauer, T. Barth, H. Helbig, M. G. A. Abdelfatah, T. Fuchsluger, M. Alami Quali, A. Decker, M. Ladewig, S. Krawczyk, K. Lenhard, B. Lackner, F. Gekeler, A. Rickmann, P. Szurmann, C. J. Gassel, N. Fischer, D. A. Wenzel, I. Seitz, L. Wolfram, K. U. Bartz-Schmidt, C. Elhardt, S. Arrow, D. S. Langhans, S. König, C. M. Wertheimer, A. Wolf, S. Dithmar, G. Knopf, A.-K. Regensburger, S. Kuehnel, D. Kampik, J. Hillenkamp

**Affiliations:** 1 Department of Ophthalmology, Ernst-Von-Bergmann Hospital, Potsdam, Germany; 2 Department of Ophthalmology, Brandenburg Medical School Theodor Fontane (MHB), University Hospital Brandenburg, Brandenburg/ Havel, Germany; 3 Department of Ophthalmology, Medical Center, Faculty of Medicine, University of Freiburg, Freiburg im Breisgau, Germany; 4 Rotterdam Eye Hospital, Rotterdam, The Netherlands

**Keywords:** Firework induced eye injuries, Firework inflicted eye injuries, Consumer fireworks, Private fireworks, Incidence, Protection, Bystander

## Abstract

**Background:**

After 2 years of pandemic sales ban, on New Year`s Eve 2022/23 consumer firework articles were officially available again in Germany and the Netherlands.

**Methods:**

In the Netherlands we prospectively and anonymously collected data on patients treated for firework induced eye injuries from 2009 on, in Germany since 2016.

**Results:**

Around New Year ´s Eve 2022/23 the number of patients with firework inflicted eye injuries increased in the Netherlands to 133 and in Germany to 838. In both countries the participation of eye departments was 90%. The incidence of firework induced eye injuries in the Netherlands was 0,8 /100 000 in 2022/23, in Germany 1,0 /100 000 in 2022/23. Comparing age groups of minors with firework induced eye injuries, in the Netherlands, total numbers of children below 12 years was lower than total numbers of adolescents between 12 and 17 years. Yet, in Germany from 2016 on every year, total number of children below 12 years were higher than total number of adolescents. The number of patients who reported on being injured as bystander was between 34% in 2020/21 and 53% in both countries.

**Conclusions:**

While the incidence of firework induced eye injuries in the Netherlands was reduced due to awareness campaigns and regulatory work, the incidence was increasing on the first New Year`s Eve after the pandemic regulations of consumer fireworks in Germany. Especially young children below 12 years need more protection in Germany. In both countries, effective measures of protection need to be implemented for protection of bystanders, whose numbers were low during the first pandemic years.

**Key messages:**

*What is known:*

- overrepresentation of affected minors below 18 years and a rate of up to 50% of injured bystanders according to our data collection over 15 years in the Netherlands and 7 years in Germany

*What is new:*

- In 2022/2023, total numbers of patients with fire work induced eye injuries in the Netherlands and Germany increased significantly after 2 years of pandemic regulations with a sales ban of consumer fire work articles
- In 2022/23, incidence of firework induced eye injuries is 1/100 000 in Germany, 0,8/100 000 in the Netherlands
- Among affected minors in Germany school children up to 12 years are at higher risk than adolescents

**Supplementary Information:**

The online version contains supplementary material available at 10.1007/s00417-024-06677-6.

## Introduction

Wherever private fireworks are operated, incidents leading to eye injuries occur [1–6]. On the other hand, global initiatives as the Vision 2020 program are aimed at reducing the number of avoidable visual impairments and blindness [7]. In an attempt to quantitate the relevance of firework induced eye injuries in the Netherlands and in Germany, we have introduced nationwide studies to prospectively collect standardized data from all patients who were treated with firework induced eye injuries in hospital related emergency services on the days around New Years´ Eve.

We report on the total number**s** of patients with firework-induced eye injuries in both countries for the past 15 years in the Netherlands and the past 7 years in Germany.

In previous publications we have documented bystanders accounted for roughly half of patients and that children and adolescents with a mean age of 11 years accounted for up to 40 percent of patients with fire work induced eye injuries [8–10]. Regarding data on age and activity, we have to ask, if regulations aimed at protecting the two most vulnerable patient groups are sufficient in the Netherlands and in Germany.

During the New Year´s season 2020/21 and 2021/22 in Germany and the Netherlands a countrywide ban of consumer fireworks was realized in order to reduce the additional treatment burden of hospitals during the SarsCov19 pandemic. The number of firework-induced eye injuries consequently decreased significantly in Germany [10] and in the Netherlands (www.Vuurwerkmanifest.nl). However, when this ban was lifted for the 2022/23 season, German and Dutch streets reverberated again under the explosions of consumer fireworks. By analyzing the total numbers of patients with fire work induced eye injuries and incidences over the years we are want to discuss strategies of prevention aimed at reducing visual impairment and blindness due to fireworks to minors and bystanders.

## Methods

Data for this descriptive study was prospectively collected with a web based standardized questionnaire that was supplied to all participating eye hospitals with emergency service during new year´s Season (December 28^th^ and January 3^rd^) in Germany and in the Netherlands. Included were all hospital based ophthalmic emergency units who responded favorably to our email invitation.

In Germany anonymous answers to questions concerning patients´ age and sex were recorded as well as the activity during the accident (self-infliction, bystander or unclear), bilaterality as well as the mode of treatment (medical or surgical / outpatient or inpatient) that served as an indicator for the severeness of the injury. On the basis of the initial findings the examiners were asked to give a prognosis of visual function. All answers were optional and as a consequence all ratios were related to the total numbers of answers to a certain item. There were no exclusion criteria.

Data on initial visual acuity were not recorded for practical reasons and post treatment visual results were not documented in order to protect anonymity.

In the Netherlands data on patients´ age and role during the incident were documented as well as prognostic factors concerning expected loss of visual acuity and the rate of primary enucleations or eviscerations.

Documentation of data was initiated in the Netherlands in 2008/09 and in Germany in 2016/17 and continuously repeated every season. As the design was only minimally adopted over the time, the total numbers of patients with firework inflicted eye injuries can be followed over time, as well as the rate of affected minors and injured bystanders and patients with severe injuries leading to treatment as inpatients or visual loss. The data of both countries can be compared and analyzed regarding the current national regulations for private fireworks valid during the very season.

Minors were defined as having an age of less than 18 years at the time of incidence. The group of minors was divided into children below 12 years of age and adolescents from 12 to 17 years of age. Bystanders are persons who reported on having been injured by a third party or in an unclear situation as opposed to `having actively ignited the fire work product`.

## Results

### Total number of injured persons and participating eye hospitals in the Netherlands

**Fig. 1:**
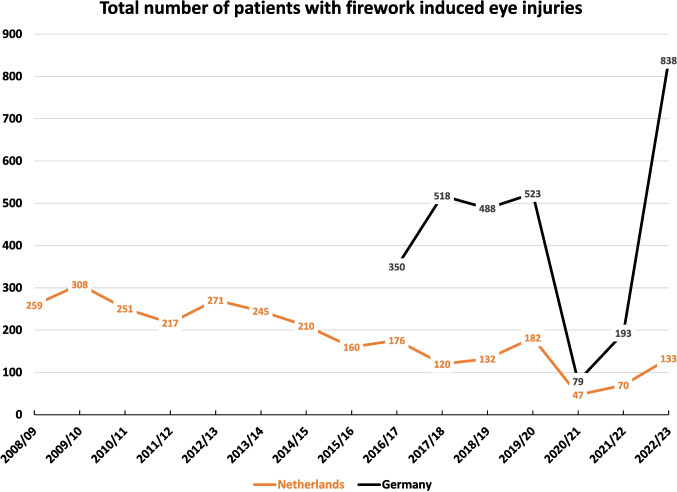
Total number of patients treated in eye departments for firework induced eye injuries per year around New Year`s Eve in the Netherlands and Germany. In the Netherlands, constant campaining and regulatory reduction of consumer firework operating time have led to a decrease of eye injurie numbers. In 2020/21 and 2021/22 a sales ban of consumer fireworks was effective in both countries in reducing eye injuries during the pandemic

The total numbers of patients with firework induced eye injuries have decreased in the Netherlands over the years from 259 in 2008/09 with a peak of 308 patients in 2009/10 to 133 in 2022/23. The two pandemic years 2020/21 and 2021/22 with a sales ban of consumer fireworks have led to a decrease to 47 and 70 patients fig. 1.

In the Netherlands participation was roughly 95% of all eye departments in hospitals with emergency service during the days around New Year´s eve.

### Total numbers of injured persons and participating eye hospitals in Germany

The total number of patients with firework induced eye injuries documented in our nationwide German study increased in the first four years. Having started with 350 patients in 2016/17, the numbers increased to 518, 488 and 523 from 2017–2020. During the two pandemic years with the sales ban of consumer fireworks and measures of social distancing the numbers decreased to 79 in 2020–2021 and to 193 in 2021–2022. In 2022/23 the total number of patients with firework-induced eye injuries increased to a maximum of 838 Figure 1.

In Germany, the number of participating hospital-linked eye emergency services could be doubled from 41 in 2016/17 to 83 in 2022/23 by personal communication and increasing awareness for the study. For the last New Year´s Eve this corresponds to a participation rate of 90% of all German eye departments with an emergency service. All 39 university eye departments as well as 55 non-university eye departments have participated.

### Incidence in The Netherlands and in Germany

In order to correct for the total number of inhabitants in each country, we calculated the incidence of firework induced eye injuries per 100 000 inhabitants based on the actual number of inhabitants (in the Netherlands ranging from 16,6 million in 2009 to 17,7 million in 2022 and in Germany from 82,5 million in 2016 to 84,3 million in 2022).

**Fig. 2:**
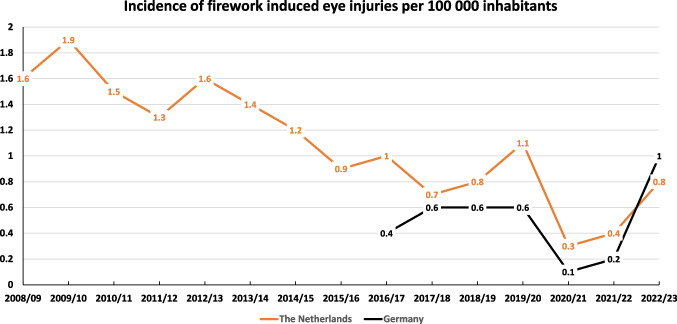
Incidence of firework induced eye injuries per 100 000 inhabitants. The incidence in Netherlands, where documentation has started in 2008/09, has constantly been reduced, whereas the incidence in Germany has surpassed the Netherlands´ in 2022/23

In the Netherlands the incidence was 1,9 per 100 000 in 2009/10 and 0,3 per 100 000 in the first pandemic year 2020/21. In Germany the incidence was lower and ranged from 0,6 per 100 000 during all years with a sufficient coverage to 1,0 per 100 000 in 2022/23, the first year after pandemic regulations Figure 2.

### The total number of minors by two age groups and severity of injuries

#### in The Netherlands

**Fig. 3:**
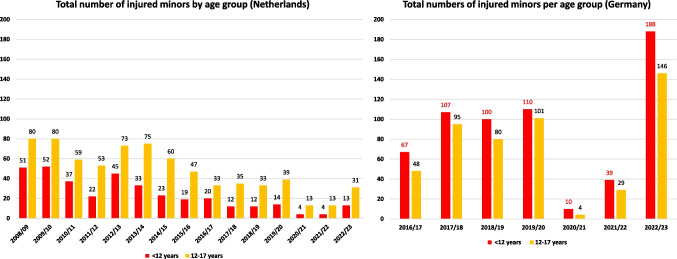
Total number of children and adolescents with firework induced eye injuries in the Netherlands and in Germany. Since 2008/09 in the Netherlands, the number of injured children under 12 years was lower than the adolescents´. In Germany in all years more children under 12 years were injured than adolescents in all years since 2016/17

During the past 15 years in the Netherlands 2781 patients with firework induced eye injuries have been documented. Overall 39% percent (1085 in total number) were minors below the age of 18. Roughly one third of these (361) patients were younger than 12 years and 724 injured adolescents (26%) were 12–17 years old (Fig. 3). 20,5% (74/361) of patients younger than 12 years were documented with an irreversible partial or complete visual loss whereas 34% of adolescent patients (246/724) accounted for irreversible partial or complete visual loss.

#### In Germany

During the past 7 years in Germany, 1124 minors below age of 18 were injured, 31,3% of all total 3589 patients, among them 621 children younger than 12 years and 503 adolescents from 12 to 17 years (Fig. 3).

One third of all minors, in total 194 patients of both age groups were treated as inpatients for severe eye injuries, 91 children under the age of 12 and 103 adolescents between 12 and 17 years.

24 minors sustained a ruptured globe, 15 of them younger than 12 years. 31 further children of the young age group were expected to be left with visual loss from the injury. During the highly explosive 2022/23 season, 3 of 4 minors with a ruptured globe were younger than 12 years.

In the age group of 12–17 years we documented 503 minors with firework induced eye injuries, among those 103 (21%) were severely injured and needed medical or surgical inpatient treatment. 9 adolescents had a ruptured globe, one sustained a severe injury with an intraocular foreign body and 35 further patients were documented with an expected visual loss.

The rate of affected minors in Germany ranged from 33 to 40% of all patients in all years except in 2020/21 when it decreased to 25%. During the 7 years of documentation the mean age of affected minors ranged consistently between 10 and 11 years. The youngest patient with firework induced eye injuries documented in Germany was a 9 days old newborn with a burn of both eyes´ lids, conjunctiva, cornea and a grade two burn of the face´s skin.

The analyzed numbers refer to patients. In the prior years of our German study 17–21% of minors had a bilateral injury. During the 2022/23 season, 77 (of 323 with data on that item) minor patients (23,8%) sustained a bilateral injury, 39 of them younger than 12 years. 15 of these young patients were treated as inpatients, whereas 12 inpatients with bilateral injuries were in the group of 12–17 years old minors. In the Dutch study between 16,2% to 64% of all patients sustained bilateral injuries.

### Total number of bystanders in the Netherlands

The total number of bystanders was 1300 over all the 15 years of documentation (2008 to 2023). 471 of theses bystanders were minors and 829 adults. 28,1% of these minors injured by a third party were severely injured, indicating that a sustained visual loss was expected by the treating ophthalmologist. The rate of bystanders ranged from a minimum of 34% in the first pandemic year and a maximum of 58% in the first year of documentation.

### Injured bystanders in Germany

**Fig. 4:**
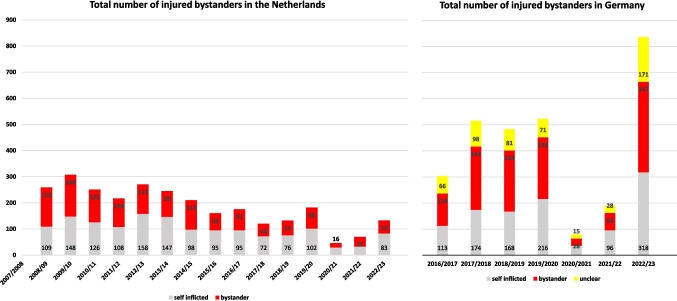
Total number of bystanders with firework induced eye injuries in the Netherlands and in Germany. The number of bystanders was decreased in the Netherlands over all years of documentation. In Germany the number of bystanders or persons, who were injured in an unclear situation was increasing over time of documentation and accounted for roughly half of all fire work induced eye injuries. If patients were added who `were injured in an unclear situation´ this rate increased up to 66%. This answer option was available in Germany only

The rate of patients in Germany who reported on having been injured as bystander ranged from 35% in the two pandemic years to 48% in all other years. The total number of documented injured bystanders in Germany was 1278 over the past seven years Figure 4.

Differing from the Dutch survey, `having been injured in an unclear situation` was an answer option in our German study. Thus, we presume this total number of bystanders slightly underestimated and increase up to 66% if we analyzed the data of `bystander´ together with `being injured in an unclear situation`.

In both countries, the female to male ratio in patients of all age groups was roughly 25% to 75% in both countries over all years, similar to previous publications.

## Discussion

### Decreasing total numbers of patients in the Netherlands, increasing numbers in Germany

The total numbers of firework induced eye injuries have evolved over the years closely related to the legislative regulations and protective actions taken in both countries.

In the Netherlands total numbers of patients have decreased over the years, apparently due to stricter rules like a ban on baby crackers, Roman candles, rockets and crackers, reduced hours of permission to use fireworks and firework free areas and public information for example by the Vuurwerkmanifest.

In Germany between 2017 and 2020 we have seen stable total numbers of roughly 500 affected patients per year.

**Fig. 5:**
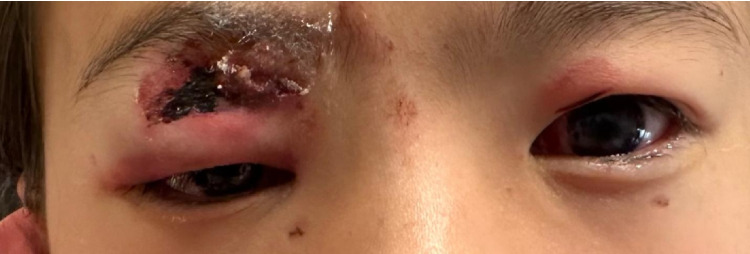
10 years old girl who was hit by a rocket as a bystander. She suffered from an upper lid injury and a severe blunt eye trauma with choroidal rupture. Resulting Snellen visual acuity was 0,1 in the right eye

The pandemic regulations with a sales ban of consumer fireworks 2020–2022 have most effectively reduced the total numbers of patients in both countries to roughly 85% of pre-pandemic total numbers. This rate is very much in accordance to the 87% reduction of firework induced eye injuries in regions with restrictive firework regulation compared to non-restrictive regulations that has been shown in the literature review by Wisse et al. [11]. The pandemic sales ban also had a positive effect on minors´ protection as shown by a decrease of minor to total number of patients ratio in both countries. After the pandemic years, however, the number and the severity of injuries in minors, reflected by bilaterality and hospital admission rates has increased Figure 5.

Germany´s increase of firework induced eye injuries in 2022/23 is alerting to us and made us have a closer look on the statistic relevance of these injuries and the involved persons: minors and bystanders.

Incidence numbers correct for the number of inhabitants and thus give an orientation on the individual risk. Yet, incidences rely on many variables for example rate of hospitals participating reflecting nationwide coverage and the number of inhabitants. The participation of all eye departments in the Netherlands was nearly 90% in all years, whereas the participation of German eye departments was steadily increased in the first years of the study. Yet, in all years with a less than 80% participation rate, due to technical reasons we have missed mostly no-patient-showed-up answers, so incidences might not have been remarkably influenced. In this analysis we have calculated the incidences per year with the actual number of inhabitants.

In 2022/23 the German incidence of fire work induced eye injuries exceeded the Dutch for the first time, indicating that regulations and actions aimed at protecting the inhabitants might be more effective in the Netherlands than in Germany. As a consequence, we are working on establishing a similarly effective alliance through different medical and social societies for Germany that ideally should be comparable to the Vuurwerkmanifest in The Netherlands.

### Higher numbers of young affected minors in Germany compared to the Netherlands

Numerous international publications for e.g. from the US [3, 12–14], China [4], India [15], Switzerland [16] and Saudi Arabia [17] have documented, that minors are clearly overrepresented among patients with firework induced eye injuries.

Besides the difficulties of examination, treatment and follow up, firework induced eye injuries to young minors imply the risk for the development of amblyopia. We have thus divided the data of affected minors into two age groups and compared the Netherlands´ data to Germany´s.

In Germany during all the past seven years, the total number of minors younger than 12 years of age was higher than the number of affected adolescents between 12 and 17 years. Compared to the Netherlands, where minors under 12 years accounted for the minority of all injured minors during the past 15 years that is a fundamental difference.

As in Germany children under 12 years are not at all allowed to use any private firework products other than category 1 devices [18] we wonder how young children get access to fireworks and if regulations were followed consequently enough to prevent young children from harm.

Due to our study desing we cannot give a complete answer, but eventual examiner notes on the answer form indicate a portion of eye injuries in young children caused by remnants of fireworks picked up. Two easy strategies to reduce these injuries would be: Every active fire work operator should clear up firework remnants immediately and children should be comprehensively instructed. Yet, apparently, children younger than 12 years in Germany seem to have access to fire work devices in a significant portion of incidents.

Referring to the data of the two pandemic years, a sales ban appears to be the most effective method of reducing the volume of fire work articles to be handled by private hands. Recent publications from India [19] and Hawaii [20] also show this effect.

Such a regulation would reduce the high risk of lifelong accumulated risk of permanent visual impairment through scar tissue development of lids, conjunctiva or cornea, loss of accommodation in lens injuries after cataract surgery, secondary glaucoma, posttraumatic retinal detachment with eventual proliferative vitreoretinopathy, retinal pigment epithelium and/or choroidal ruptures in minors and young adults [2, 21–24]. Our study does not include follow up or visual results, yet several studies have documented a high risk for visual impairment after severe eye injuries [6, 16, 25–27].

As eye injuries account for roughly 20% of all injuries caused by fireworks [3, 12], hand injuries [28], head-, neck [29] and bang traumas [30] could also significantly be reduced by such regulations.

### Almost half of the patients are bystanders

**Fig. 6:**
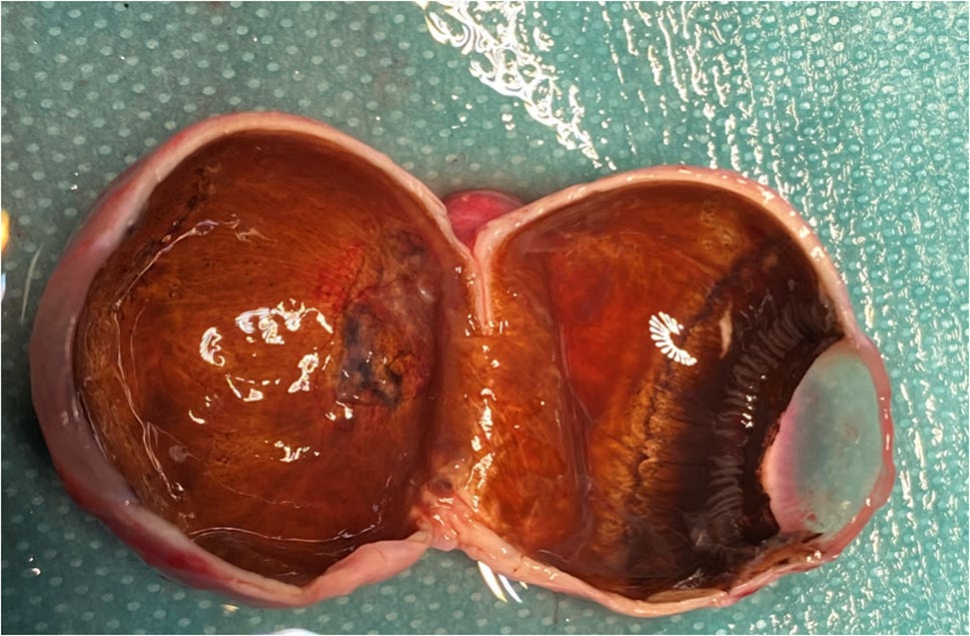
Enucleated globe of a bystander, who sustained a severe blunt trauma with total loss of iris and lens into the vitreous by an unknown consumer firework on New Year´s Eve 2022/23. Recurrent retinal detachments due to proliferative vitreoretinopathy (PVR) and a painful secondary glaucoma led to the total loss of vision and an enucleation within 6 months from the incident

With a height range of up to 100 m, rockets can cause injuries far from the site of ignition. The high number of injured bystanders is alerting. We have documented 1278 injured bystanders in Germany and 1300 affected bystanders in The Netherlands. Spectators or passers-by who were at the wrong place at the wrong time and involuntarily hit by an unidentifiable firework article (Fig. 6).

A very tragic case in Germany: A janitor collecting the leftovers of a third party´ s firework, who was severely injured in one eye and was expected to be left with a severe visual limitation. One fourth, in numbers 320 bystanders, were severely injured in Germany during the past 7 years and sustained a high risk of visual loss. Beside the psychological effect of an injury that limits visual acuity, these patients usually have limited possibilities to sue the responsible operator for legal or financial consequences.

## Conclusions

With the 15 years´ data of the Netherlands and the 7 years data of Germany, we have shown the relevance of firework induced eye injuries in both countries.

While the total number of these avoidable injuries was reduced in the Netherlands during the last 15 years by the work of the Vuurwerkmanifest, in Germany the total number of injuries has reached a maximum in the first year after the pandemic sales ban of private firework articles. A campaign for increasing the safety is necessary, especially to prevent young children and bystanders from severe eye injuries. Safe fireworks from professionals rather than from private hands should be promoted.

## Supplementary Information

Below is the link to the electronic supplementary material.

Supplementary file1  (DOCX 22.6 KB)
