# Correction: Significant increase of firework induced eye injuries in Germany and The Netherlands- are we doing enough to protect minors and bystanders?

**DOI:** 10.1007/s00417-025-06970-y

**Published:** 2025-10-16

**Authors:** Ameli Gabel-Pfisterer, Stefan Johann Lang, Daniel Boehringer, Hansjürgen Agostini, Lotte C. de Geus, Jan Tjeerd de Faber, the German firework study group, M. Fuest, P. Walter, I. Winkelmann, K. Hartmann, C. Kojetinsky, A Mueller, C. Dempe, N. Al-Ashi, H. Breuß, I. Seibel, M. Gutmann, T. Bonaventura, O. Zeitz, B. Müller, A. Joussen, P. Schwarz, Ch. Wirbelauer, H. Hofmayer, J. Wachtlin, N. I. Steinhorst, J. F. Meyer, B. Acar, S. Aisenbrey, K. Goebel, P. Rieck, M. Shalabi, M. Tohami, E. C. Thieme, A. Walch, J. Verbeck, M. Alnawaiseh, T. Schultz, N. Tsiampalis, J. Rehmann, U. Sliwowska, M. Schojai, K. Schulze, N. Kamguia, C. Wirtz, B. H. Dick, Y. Liermann, F. M. Schuetzeichel, D. Voelcker, M. Wintergerst, M. Pfau, C. Melzer, D. Hoegen, F. Bosch, J. C. Andresen, T. Krohne, F. Holz, A. Alahmad, M. Kathke, A. Sturm, E. Chankiewitz, O. Kemper, A. Heider, B. Erdogan-Uelker, N. Krainau, M. Thiele, S. Brandtner, J. Hecker, P. Strassburger, K. Engelmann, F. Lehmann, U. Brunner, H. Sachs, S. Koerner, L. Grajewski, L. Krause, W. Bayoudh, B. Yanar, D. Mpoutsis-Voutsis, J. Gerhardt, K. Rüdiger, T. Böker, T. Hejna, J. Hejna, K. Kiel, B. Breuer, E. Matthe, D. Sandner, L. Pillunat, J. Prueß-Hölscher, R. Kourukmas, M. Cieplucha, G. Geerling, R. A. Widder, G. Roessler, K. Bronikowska, M. Thomalla, F. Filev, B. von Jagow, J. Walther, C. Zollfrank, M. Blum, T. Theofilos, Y. M. Christian, F.E. Kruse, A Foerster, I. Diamantis, C. Braun, T. Kiefer, P. Rating, M. Fiorentzis, N. E. Bechrakis, A. Scheider, K. P. Kaiser, M. L. Biller, J. Bucur, T. Arad, M. Müller, T. Kohnen, J. Grabs, U. Vossmerbaeumer, C. Puk, Y. Laich, T. Reinhard, J. Quandt, A. Lieder, J. Seewald, C. Mais, R. E. Carlos, M. H. Graef, M. Rehak, J. Schrecker, Ute Just, C. Schinzel, S. T. Meyer, N. Feltgen, H. Hoerauf, T. Trzos, I. Prusiecki, M. C. Bründer, S. Paul, A. Stahl, R. Wienrich, A. Viestenz, A. Huth, A. Viestenz, A. Schulz, B. Fuisting, L. Mautone, A. Özen, N. Kaupke, L. Kröger, M. Alsarrani, I. Lau, J. Birtel, F. Hagenau, J. Wildner, N. Kounatidou, A. Hassenstein, C. Skevas, V. Knospe, S. Vardanyan, C. Grohmann, M. Spitzer, L. Fuhrmann, M. Schargus, M. T. Eddy, D. Rose, K. Reinkemeier, L. Armonies, B. Stemplewitz, U. Schaudig, A. Scheuerle, M. Auerbach, C. Beisse, K. Rohrschneider, R. Khoramnia, G. Auffahrt, N. Mala, L. Hesse, A. Sneyers, P. Kohlhas, E. Flockerzi, F. N. Fries, L. Daas, B. Seitz, R. Augsten, M. Aghi, M. Zankel, A. Ghaith, S. Weber, U. Voigt, D. Meller, J. O. Rudolph, M. Müller, F. Brede, M. Alia, F. Treumer, M. Saeger, B. Nölle, C. Ehlken, J. B. Roider, A. Hueber, C. Cursiefen, P. Esser, M. Kroeger, N. Viehweg, M. Knorr, P. Meier, C. Girbardt, C. Bormann, N. Suckert, J. Letzel, F. Ziemssen, V Pawlik, C Schiemenz, M Busch, P. Schubart, R. Piria, M. Stöcker, A. M. Mohi Sefat, F. Rommel, S. Grisanti, I. Bastron, M. Benthami, D-N. Roman, S. Kaskel-Paul, C. Argyrios, A. Agharza, K. Bermond, M. A. Strobel, L. O. Hattenbach, M. Erwemi, F. Schlichtenbrede, B. Stoffelns, A. Schuster, N. Pfeiffer, C. Paul, W. Sekundo, G. Renieri, H. Thieme, P. Foerster, S. Priglinger, E. Von Koskull, M. Maier, F. Alten, N. Eter, K. C. Brinkmann, F. Alshikh, V. Klishko, U. Holland, A. Medra, A. Weber, H. Höh, F. Luciani, J. Schmidbauer, A. Pielen, P. C. Horn, K. Hille, S. Grafmueller, G. Esper, T. Ahmels, L. Kolbeck, P. Kupper, A-S. Schröder, F. Keller, S. Schrader, Höhn Fabian, M. G. Häringer, J. Schiemann, A. Liekfeld, M. Dütsch, V. Schnitzbauer, T. Barth, H. Helbig, M. G. A. Abdelfatah, T. Fuchsluger, M. Alami Quali, A. Decker, M. Ladewig, A. Rickmann, P. Szurmann, C. J. Gassel, N. Fischer, D. A. Wenzel, I. Seitz, L. Wolfram, K. U. Bartz-Schmidt, C. Elhardt, S. Arrow, D. S. Langhans, S. König, C. M. Wertheimer, A. Wolf, S. Dithmar, G. Knopf, A-K. Regensburger, S. Kuehnel, D. Kampik, J. Hillenkamp

**Affiliations:** 1 Department of Ophthalmology, Ernst-Von-Bergmann Hospital, Potsdam, Germany; 2 Department of Ophthalmology, Brandenburg Medical School Theodor Fontane (MHB), University Hospital Brandenburg, Brandenburg/ Havel, Germany; 3 Department of Ophthalmology, Medical Center, Faculty of Medicine, University of Freiburg, Freiburg im Breisgau, Germany; 4 Rotterdam Eye Hospital, Rotterdam, The Netherlands

**Correction: Graefe's Archive for Clinical and Experimental Ophthalmology (2024) 263:1157-1165**

**https://doi.org/10.1007/s00417-024-06677-6**

In the published original article, the collaborators are missing.

The collaborators of the study group are listed below:

1. Fuest M., Walter P., Department of Ophthalmology, RWTH Aachen University, Aachen, Germany
2. Winkelmann I., Hartmann K., Kojetinsky C., Mueller A., University Hospital Augsburg, Department of Ophthalmology, Augsburg
3. Dempe C., Al-Ashi N., Department of Ophthalmology, Oberlausitz Hospitals gGmbH Hospital Bautzen, Bautzen, Germany
4. Breuß H., Seibel I., Department of Ophthalmology, Helios Klinikum Berlin-Buch, Berlin Germany
5. Gutmann M., Bonaventura T., Zeitz O., Müller B., Joussen A., Charité—Universitätsmedizin Berlin, Corporate Member of Freie Universität, Campus Rudolph-Virchow and Campus Benjamin-Franklin, Berlin, Germany
6. Schwarz P., Wirbelauer Ch., Eyeclinic Berlin-Marzahn GmbH, Berlin, Germany
7. Hofmayer H.^1^, Wachtlin J.^1,2^, ^1^ Department of Ophthalmology, St. Gertrauden Krankenhaus Berlin, Berlin, Germany, ^2^ Brandenburg Medical School Theodor Fontane (MHB), Neuruppin, Germany
8. Steinhorst N. I., Meyer J. F., Acar B., Aisenbrey S., Department of Ophthalmology, Vivantes Health Network, Neukoelln Hospital, Berlin, Germany
9. Goebel K., Rieck P., Department of Ophthalmology, Schlosspark Hospital, Berlin, Germany
10. Shalabi M., Tohami M., Thieme E.C., Walch A., Verbeck J., Alnawaiseh M., Department of Ophthalmology, Klinikum Bielefeld Gem. GmbH, Bielefeld, Germany.
11. Schultz T., Tsiampalis N., Rehmann J., Sliwowska U., Schojai M., Schulze K., Kamguia N., Wirtz C., Dick B.H., Ruhr University Eye Hospital Bochum, Bochum, Germany
12. Liermann Y., Schuetzeichel F.M., Voelcker D., Wintergerst M., Pfau M., Melzer C., Hoegen D., Bosch F., Andresen J.C., Krohne T., Holz F., Department of Ophthalmology, University of Bonn, Bonn, Germany
13. Alahmad A., Kathke M., Sturm A., Department of Ophthalmology, University Hospital Brandenburg, Brandenburg Medical School Theodor Fontane (MHB), Brandenburg an der Havel, Germany
14. Chankiewitz E., Department of Ophthalmology, Academic Hospital Braunschweig, Braunschweig, Germany
15. Kemper O., Heider A., Erdogan-Uelker B., Krainau N., Thiele M., Brandtner S., Department of Ophthalmology, Klinikum Bremen Mitte, Bremen, Germany
16. Hecker J., Strassburger P., Engelmann K., Klinikum Chemnitz gGmbH, Ophthalmic Clinic Chemnitz, Germany
17. Lehmann F., Brunner U., Sachs H., Department of Ophthalmology, Carl-Thiem-Klinikum Cottbus, Cottbus, Germany
18. Koerner S., Grajewski L., Krause L., Department of Ophthalmology, Staedtisches Klinikum Dessau, Brandenburg Medical School Theodor Fontane, Dessau, Germany
19. Bayoudh W., Yanar B., Mpoutsis-Voutsis D., Gerhardt J., Rüdiger K., Böker T., Department of Ophthalmology, Municipal Hospital of Dortmund, Dortmund, Germany
20. Hejna T., Hejna J., Kiel K., Breuer B., Department of Ophthalmology, Hospital Dresden-Friedrichstadt, Dresden, Germany
21. Matthe E., Sandner D., Pillunat L., Department of Ophthalmology, University Hospital Carl Gustav Carus, TU Dresden, Germany
22. Prueß-Hölscher J., Kourukmas R., Cieplucha M., Geerling G., Department of Ophthalmology, Medical Faculty and University Hospital Düsseldorf, Germany,
23. Widder R. A., Roessler G., Department of Ophthalmology, St. Martinus-Krankenhaus, Düsseldorf, Germany
24. Bronikowska K., Thomalla M., Department of Ophthalmology, Evangelisches Krankenhaus Duisburg, Germany
25. Filev F., von Jagow B., Augenklinik Klinikum Barnim, Department of Ophthalmology, Werner Forßmann Klinikum, Eberswalde, Germany
26. Walther J., Zollfrank C., Blum M., Department of Ophthalmology, Helios Clinic Erfurt, Erfurt, Germany
27. Theofilos T., Christian Y.M., Friedrich E Kruse, Department of Ophthalmology, University of Erlangen-Nürnberg, Erlangen, Germany
28. Foerster A., Diamantis I., Braun C., Kiefer T., Rating P., Fiorentzis M., Bechrakis N. E., Department of Ophthalmology, University Hospital Essen, Essen Germany
29. Scheider A., Department of Ophthalmology, Evangelisches Krankenhaus Essen-Weerden, Essen, Germany
30. Kaiser K. P., Biller M. L., Bucur J., Arad T., Müller M., Kohnen T., Department of Ophthalmology, Goethe-University, Frankfurt, Germany
31. Grabs J., Vossmerbaeumer U., Department of Ophthalmology, Frankfurt Hoechst Eye Hospital, Frankfurt/Mai, Germany
32. Puk C., Department of Ophthalmology, Klinikum Frankfurt/Oder GmbH, Frankfurt/Oder, Germany
33. Laich Y., Reinhard T., Eye Center, Medical Center - University of Freiburg, Faculty of Medicine, University of Freiburg, Germany
34. Quandt J., Lieder A., Seewald J., Department of Ophthalmology, SRH Waldklinikum Gera, Gera, Germany
35. Mais C., Carlos R. E., Graef M. H., Rehak M., Department of Ophthalmology, Justus-Liebig-University Giessen, Giessen, Germany
36. Schrecker J., Just Ute, Eye hospital Glauchau, Glauchau, Germany
37. Schinzel C., Meyer S. T., Feltgen N., Hoerauf H., Department of Ophthalmology, Georg-August University Göttingen, Göttingen, Germany
38. Trzos T., Prusiecki I., Department of Ophthalmology, Hospital Goerlitz, Goerlitz, Germany
39. Bründer M.C., Paul S., Stahl A., Department of Ophthalmology, University Medicine Greifswald, Greifswald, Germany
40. Wienrich R., Viestenz A., Huth A., Viestenz A., Department of Ophthalmology, University Medicine Halle, Martin-Luther-University Halle-Wittenberg, Halle/Saale, Germany
41. Schulz, A. Fuisting B., Mautone L., Özen A., Kaupke N., Kröger L., Alsarrani M., Lau I., Birtel J., Hagenau F., Wildner J., Kounatidou N., Hassenstein A., Skevas C., Knospe V., Vardanyan S., Grohmann C., Spitzer M., University Medical Center Hamburg-Eppendorf, Department of Ophthalmology, Hamburg, Germany
42. Fuhrmann L., Schargus M., Department of Ophthalmology, Asklepios Hospital Nord-Heidberg, Hamburg, Germany
43. Eddy M.T., Rose D., Department of Ophthalmology, Asklepios Hospital Altona, Hamburg, Gerrmany
44. Reinkemeier K., Armonies L., Stemplewitz B., Schaudig U., Department of Ophthalmology, Asklepios Hospital Barmbek, Hamburg, Germany
45. Book B., Hufendiek K., Panidou-Marschelke E., Sinicin E., Lindziute M., Rauscher J. T. R., Hamann M., Framme C., Department of Ophthalmology, Medical University Hannover, Hannover, Germany
46. Scheuerle A., Auerbach M., Beisse C., Rohrschneider K., Khoramnia R., Auffahrt G., Department of Ophthalmology, University Heidelberg, Heidelberg, Germany
47. Mala N., Hesse L., Department of Ophthalmology, SLK-Hospital Heilbronn GmbH, Heilbronn 22/23
48. Sneyers A., Kohlhas P., Flockerzi E., Fries F.N., Daas L., Seitz B., Department of Ophthalmology Saarland University Medical Center, Homburg, Germany
49. Augsten R., Aghi M., Zankel M., Ghaith A., Weber S., Voigt U., Meller D., Department of Ophthalmology, University of Jena, Jena, Germany
50. Rudolph J.O., Müller M., Brede F., Alia M., Treumer F., Department of Ophthalmology, Klinikum Kassel, Kassel, Germany
51. Saeger M., Nölle B., Ehlken C., Roider J. B., Department of Ophthalmology, University of Schleswig-Holstein Kiel, Kiel, Germany.
52. Hueber A., Cursiefen .C, Dept. of Ophthalmology, Medical Faculty, University of Cologne, Köln, Germany
53. Schrage N., Department of Ophthalmology, Hospitals of the City of Köln, Köln-Merheim, Köln, Germany
54. Esser P., Augenklinik, Department of Ophthalmology, St. Elisabeth-Hopsital, Köln-Hohenlind, Köln, Germany
55. Kroeger M., Viehweg N., Knorr M., Department of Ophthalmology, Helios Hospital Krefeld, Krefeld, Germany
56. Meier P, Girbardt C., Bormann C., Suckert N., Letzel J., Ziemssen F, University Eye Hospital, Leipzig University, Germany.
57. Pawlik V, Schiemenz C, Busch M, Schubart P., Piria R., Stöcker M., Mohi Sefat A.M., Rommel F., Grisanti S., Department of Ophthalmology, University of Schleswig-Holstein Lübeck, Lübeck, Germany
58. Bastron I., Benthami M., Roman D-N., Kaskel-Paul S., Department of Ophthalmology, Klinikum Lüdenscheid, Märkische Kliniken GmbH,
59. Argyrios C., Agharza A., Bermond K., Strobel M. A., Hattenbach L.O., Department of Ophthalmology, Hospital of Ludwigshafen, Ludwigshafen am Rhein, Germany
60. Erwemi M., Schlichtenbrede F., Department of Ophthalmology, University of Mannheim, Mannheim, Germany
61. Stoffelns B., Schuster A., Pfeiffer N., Department of Ophthalmology, Mainz University Medical Center, Mainz, Germany
62. Paul C., Sekundo W., Department of Ophthalmology, University Hospital Marburg, Marburg, Germany
63. Renieri G., Thieme H., Department of Ophthalmology, Otto-von-Guericke University Magdeburg, Magdeburg, Germany
64. Foerster P., Priglinger S., Department of Ophthalmology, University Hospital, Ludwig-Maximilians-University München, Munich, Germany
65. Von Koskull E., Maier M., Department of Ophthalmology, University Hospital, Technical University Munich, Munich, Germany
66. Alten F., Eter N., Department of Ophthalmology, University of Muenster Medical Center, Muenster, Germany
67. Brinkmann K.C., Alshikh F., Klishko, V., Holland, U., Medra A., Weber A., Höh H., Department of Ophthalmology, Hospital Dietrich-Bonhoeffer, Neubrandenburg, Germany
68. Luciani F., Schmidbauer J., Department of Ophthalmology, Klinikum Nürnberg Nord, Nuremberg, Germany
69. Pielen A., Maximilians-Augenklinik Nürnberg, Nuremberg, Germany
70. Horn, P. C., Hille K., Eye Hospital, Ortenau-Klinikum Offenburg, Offenburg Germany
71. Grafmueller S., Esper G., Ahmels T., Kolbeck L., Kupper P., Schröder A-S., Keller F., Schrader S., Pius Hospital Oldenburg, Hospital at Medical University Oldenburg, Oldenburg, Germany
72. Höhn Fabian, Eye Clinic, Marienhospital Osnabrück, Osnabrück, Germany
73. Häringer M.G.^1^, Schiemann J.^1^, Liekfeld A.^1,2^, ^1^Department of Ophthalmology, Hospital Ernst-von-Bergmann Potsdam, Potsdam, Germany, ^2^ University of Applied Science Brandenburg, Rathenow, Germany
74. Dütsch M., Schnitzbauer V., Barth T., Helbig H., Department of Ophthalmology, University Medical Centre Regensburg, Regensburg, Deutschland.
75. Abdelfatah M.G.A., Fuchsluger T., Department of Ophthalmology, University Medical Centre Rostock, Rostock, Germany
76. Alami Quali M., Decker A., Ladewig M., Department of Ophthalmology, Hospital Saarbrücken, Saarbrücken, Germany
77. Krawczyk S., Lenhard, K., Lackner B., Gekeler F., Department of Ophthalmology, Katharinenhospital Stuttgart, Stuttgart, Germany
78. Rickmann A., Szurmann P., Knappschaft Hospital Saar, Sulzbach/Saar, Germany
79. Gassel C. J., Fischer N., Wenzel D. A., Seitz I., Wolfram L., Bartz-Schmidt K.U., Centre for Ophthalmology, University Hospital Tübingen, Tübingen, Germany
80. Elhardt C., Arrow S., Langhans D.S., König S., Wertheimer C. M., Wolf A., Department of Ophthalmology, University Hospital Ulm, Ulm, Germany
81. Dithmar S., Department of Ophthalmology, Helios Hospital HSK Wiesbaden, Wiesbaden, Germany
82. Knopf G., Regensburger A-K., Kuehnel S., Kampik D., Hillenkamp J., Department of Ophthalmology, University Hospital Wuerzburg, Wuerzburg, Germany

The original article has been corrected.

