# Supplementary material for: Significant increase of firework induced eye injuries in Germany and The Netherlands- are we doing enough to protect minors and bystanders?
Source: Graefes Arch Clin Exp Ophthalmol. 2024 Dec 4;263(4):1157–65. doi: 10.1007/s00417-024-06677-6 (PMC12095323; doi:10.1007/s00417-024-06677-6)
Supplement: Supplementary file 1 — (DOCX 22.6 KB) [file 417_2024_6677_MOESM1_ESM.docx]

**Study group list**

1. Fuest M., Walter P., Department of Ophthalmology, RWTH Aachen University, Aachen, Germany
2. Winkelmann I., Hartmann K., Kojetinsky C., Mueller A., University Hospital Augsburg, Department of Ophthalmology, Augsburg
3. Dempe C., Al-Ashi N., Department of Ophthalmology, Oberlausitz Hospitals gGmbH Hospital Bautzen, Bautzen, Germany
4. Breuß H., Seibel I., Department of Ophthalmology, Helios Klinikum Berlin-Buch, Berlin Germany
5. Gutmann M., Bonaventura T., Zeitz O., Müller B., Joussen A., Charité—Universitätsmedizin Berlin, Corporate Member of Freie Universität, Campus Rudolph-Virchow and Campus Benjamin-Franklin, Berlin, Germany
6. Schwarz P., Wirbelauer Ch., Eyeclinic Berlin-Marzahn GmbH, Berlin, Germany
7. Hofmayer H.^1^, Wachtlin J.^1,2^, ^1^ Department of Ophthalmology, St. Gertrauden Krankenhaus Berlin, Berlin, Germany, ^2^ Brandenburg Medical School Theodor Fontane (MHB), Neuruppin, Germany
8. Steinhorst N. I., Meyer J. F., Acar B., Aisenbrey S., Department of Ophthalmology, Vivantes Health Network, Neukoelln Hospital, Berlin, Germany
9. Goebel K., Rieck P., Department of Ophthalmology, Schlosspark Hospital, Berlin, Germany
10. Shalabi M., Tohami M., Thieme E.C., Walch A., Verbeck J., Alnawaiseh M., Department of Ophthalmology, Klinikum Bielefeld Gem. GmbH, Bielefeld, Germany.
11. Schultz T., Tsiampalis N., Rehmann J., Sliwowska U., Schojai M., Schulze K., Kamguia N., Wirtz C., Dick B.H., Ruhr University Eye Hospital Bochum, Bochum, Germany
12. Liermann Y., Schuetzeichel F.M., Voelcker D., Wintergerst M., Pfau M., Melzer C., Hoegen D., Bosch F., Andresen J.C., Krohne T., Holz F., Department of Ophthalmology, University of Bonn, Bonn, Germany
13. Alahmad A., Kathke M., Sturm A., Department of Ophthalmology, University Hospital Brandenburg, Brandenburg Medical School Theodor Fontane (MHB), Brandenburg an der Havel, Germany
14. Chankiewitz E., Department of Ophthalmology, Academic Hospital Braunschweig, Braunschweig, Germany
15. Kemper O., Heider A., Erdogan-Uelker B., Krainau N., Thiele M., Brandtner S., Department of Ophthalmology, Klinikum Bremen Mitte, Bremen, Germany
16. Hecker J., Strassburger P., Engelmann K., Klinikum Chemnitz gGmbH, Ophthalmic Clinic Chemnitz, Germany
17. Lehmann F., Brunner U., Sachs H., Department of Ophthalmology, Carl-Thiem-Klinikum Cottbus, Cottbus, Germany
18. Koerner S., Grajewski L., Krause L., Department of Ophthalmology, Staedtisches Klinikum Dessau, Brandenburg Medical School Theodor Fontane, Dessau, Germany
19. Bayoudh W., Yanar B., Mpoutsis-Voutsis D., Gerhardt J., Rüdiger K., Böker T., Department of Ophthalmology, Municipal Hospital of Dortmund, Dortmund, Germany
20. Hejna T., Hejna J., Kiel K., Breuer B., Department of Ophthalmology, Hospital Dresden-Friedrichstadt, Dresden, Germany
21. Matthe E., Sandner D., Pillunat L., Department of Ophthalmology, University Hospital Carl Gustav Carus, TU Dresden, Germany
22. Prueß-Hölscher J., Kourukmas R., Cieplucha M., Geerling G., Department of Ophthalmology, Medical Faculty and University Hospital Düsseldorf, Germany,
23. Widder R. A., Roessler G., Department of Ophthalmology, St. Martinus-Krankenhaus, Düsseldorf, Germany
24. Bronikowska K., Thomalla M., Department of Ophthalmology, Evangelisches Krankenhaus Duisburg, Germany
25. Filev F., von Jagow B., Augenklinik Klinikum Barnim, Department of Ophthalmology, Werner Forßmann Klinikum, Eberswalde, Germany
26. Walther J., Zollfrank C., Blum M., Department of Ophthalmology, Helios Clinic Erfurt, Erfurt, Germany
27. Theofilos T., Christian Y.M., Friedrich E Kruse, Department of Ophthalmology, University of Erlangen-Nürnberg, Erlangen, Germany
28. Foerster A., Diamantis I., Braun C., Kiefer T., Rating P., Fiorentzis M., Bechrakis N. E., Department of Ophthalmology, University Hospital Essen, Essen Germany
29. Scheider A., Department of Ophthalmology, Evangelisches Krankenhaus Essen-Weerden, Essen, Germany
30. Kaiser K. P., Biller M. L., Bucur J., Arad T., Müller M., Kohnen T., Department of Ophthalmology, Goethe-University, Frankfurt, Germany
31. Grabs J., Vossmerbaeumer U., Department of Ophthalmology, Frankfurt Hoechst Eye Hospital, Frankfurt/Mai, Germany
32. Puk C., Department of Ophthalmology, Klinikum Frankfurt/Oder GmbH, Frankfurt/Oder, Germany
33. Laich Y., Reinhard T., Eye Center, Medical Center - University of Freiburg,

Faculty of Medicine, University of Freiburg, Germany

1. Quandt J., Lieder A., Seewald J., Department of Ophthalmology, SRH Waldklinikum Gera, Gera, Germany
2. Mais C., Carlos R. E., Graef M. H., Rehak M., Department of Ophthalmology, Justus-Liebig-University Giessen, Giessen, Germany
3. Schrecker J., Just Ute, Eye hospital Glauchau, Glauchau, Germany
4. Schinzel C., Meyer S. T., Feltgen N., Hoerauf H., Department of Ophthalmology, Georg-August University Göttingen, Göttingen, Germany
5. Trzos T., Prusiecki I., Department of Ophthalmology, Hospital Goerlitz, Goerlitz, Germany
6. Bründer M.C., Paul S., Stahl A., Department of Ophthalmology, University Medicine Greifswald, Greifswald, Germany
7. Wienrich R., Viestenz A., Huth A., Viestenz A., Department of Ophthalmology, University Medicine Halle, Martin-Luther-University Halle-Wittenberg, Halle/Saale, Germany
8. Schulz, A. Fuisting B., Mautone L., Özen A., Kaupke N., Kröger L., Alsarrani M., Lau I., Birtel J., Hagenau F., Wildner J., Kounatidou N., Hassenstein A., Skevas C., Knospe V., Vardanyan S., Grohmann C., Spitzer M., University Medical Center Hamburg-Eppendorf, Department of Ophthalmology, Hamburg, Germany
9. Fuhrmann L., Schargus M., Department of Ophthalmology, Asklepios Hospital Nord-Heidberg, Hamburg, Germany
10. Eddy M.T., Rose D., Department of Ophthalmology, Asklepios Hospital Altona, Hamburg, Gerrmany
11. Reinkemeier K., Armonies L., Stemplewitz B., Schaudig U., Department of Ophthalmology, Asklepios Hospital Barmbek, Hamburg, Germany
12. Book B., Hufendiek K., Panidou-Marschelke E., Sinicin E., Lindziute M., Rauscher J. T. R., Hamann M., Framme C., Department of Ophthalmology, Medical University Hannover, Hannover, Germany
13. Scheuerle A., Auerbach M., Beisse C., Rohrschneider K., Khoramnia R., Auffahrt G., Department of Ophthalmology, University Heidelberg, Heidelberg, Germany
14. Mala N., Hesse L., Department of Ophthalmology, SLK-Hospital Heilbronn GmbH, Heilbronn 22/23
15. Sneyers A., Kohlhas P., Flockerzi E., Fries F.N., Daas L., Seitz B., Department of Ophthalmology Saarland University Medical Center, Homburg, Germany
16. Augsten R., Aghi M., Zankel M., Ghaith A., Weber S., Voigt U., Meller D., Department of Ophthalmology, University of Jena, Jena, Germany
17. Rudolph J.O., Müller M., Brede F., Alia M., Treumer F., Department of Ophthalmology, Klinikum Kassel, Kassel, Germany

| 1. Saeger M., Nölle B., Ehlken C., Roider J. B., Department of Ophthalmology, University of Schleswig-Holstein Kiel, Kiel, Germany. |
| --- |
| 1. Hueber A., Cursiefen .C, Dept. of Ophthalmology, Medical Faculty, University of Cologne, Köln, Germany |

1. Schrage N., Department of Ophthalmology, Hospitals of the City of Köln, Köln-Merheim, Köln, Germany
2. Esser P., Augenklinik, Department of Ophthalmology, St. Elisabeth-Hopsital, Köln-Hohenlind, Köln, Germany
3. Kroeger M., Viehweg N., Knorr M., Department of Ophthalmology, Helios Hospital Krefeld, Krefeld, Germany
4. Meier P, Girbardt C., Bormann C., Suckert N., Letzel J., Ziemssen F, University Eye Hospital, Leipzig University, Germany.
5. Pawlik V, Schiemenz C, Busch M, Schubart P., Piria R., Stöcker M., Mohi Sefat A.M., Rommel F., Grisanti S., Department of Ophthalmology, University of Schleswig-Holstein Lübeck, Lübeck, Germany
6. Bastron I., Benthami M., Roman D-N., Kaskel-Paul S., Department of Ophthalmology, Klinikum Lüdenscheid, Märkische Kliniken GmbH,
7. Argyrios C., Agharza A., Bermond K., Strobel M. A., Hattenbach L.O., Department of Ophthalmology, Hospital of Ludwigshafen, Ludwigshafen am Rhein, Germany
8. Erwemi M., Schlichtenbrede F., Department of Ophthalmology, University of Mannheim, Mannheim, Germany

| 1. Stoffelns B., Schuster A., Pfeiffer N., Department of Ophthalmology, Mainz University Medical Center, Mainz, Germany |
| --- |

1. Paul C., Sekundo W., Department of Ophthalmology, University Hospital Marburg, Marburg, Germany
2. Renieri G., Thieme H., Department of Ophthalmology, Otto-von-Guericke University Magdeburg, Magdeburg, Germany
3. Foerster P., Priglinger S., Department of Ophthalmology, University Hospital, Ludwig-Maximilians-University München, Munich, Germany
4. Von Koskull E., Maier M., Department of Ophthalmology, University Hospital, Technical University Munich, Munich, Germany
5. Alten F., Eter N., Department of Ophthalmology, University of Muenster Medical Center, Muenster, Germany
6. Brinkmann K.C., Alshikh F., Klishko, V., Holland, U., Medra A., Weber A., Höh H., Department of Ophthalmology, Hospital Dietrich-Bonhoeffer, Neubrandenburg, Germany
7. Luciani F., Schmidbauer J., Department of Ophthalmology, Klinikum Nürnberg Nord, Nuremberg, Germany
8. Pielen A., Maximilians-Augenklinik Nürnberg, Nuremberg, Germany
9. Horn, P. C., Hille K., Eye Hospital, Ortenau-Klinikum Offenburg, Offenburg Germany
10. Grafmueller S., Esper G., Ahmels T., Kolbeck L., Kupper P., Schröder A-S., Keller F., Schrader S., Pius Hospital Oldenburg, Hospital at Medical University Oldenburg, Oldenburg, Germany
11. Höhn Fabian, Eye Clinic, Marienhospital Osnabrück, Osnabrück, Germany
12. Häringer M.G.^1^, Schiemann J.^1^, Liekfeld A.^1,2^, ^1^Department of Ophthalmology, Hospital Ernst-von-Bergmann Potsdam, Potsdam, Germany, ^2^ University of Applied Science Brandenburg, Rathenow, Germany
13. Dütsch M., Schnitzbauer V., Barth T., Helbig H., Department of Ophthalmology, University Medical Centre Regensburg, Regensburg, Deutschland.
14. Abdelfatah M.G.A., Fuchsluger T., Department of Ophthalmology, University Medical Centre Rostock, Rostock, Germany
15. Alami Quali M., Decker A., Ladewig M., Department of Ophthalmology, Hospital Saarbrücken, Saarbrücken, Germany
16. Krawczyk S., Lenhard, K., Lackner B., Gekeler F., Department of Ophthalmology, Katharinenhospital Stuttgart, Stuttgart, Germany
17. Rickmann A., Szurmann P., Knappschaft Hospital Saar, Sulzbach/Saar, Germany
18. Gassel C. J., Fischer N., Wenzel D. A., Seitz I., Wolfram L., Bartz-Schmidt K.U., Centre for Ophthalmology, University Hospital Tübingen, Tübingen, Germany
19. Elhardt C., Arrow S., Langhans D.S., König S., Wertheimer C. M., Wolf A., Department of Ophthalmology, University Hospital Ulm, Ulm, Germany
20. Dithmar S., Department of Ophthalmology, Helios Hospital HSK Wiesbaden, Wiesbaden, Germany
21. Knopf G., Regensburger A-K., Kuehnel S., Kampik D., Hillenkamp J., Department of Ophthalmology, University Hospital Wuerzburg, Wuerzburg, Germany
